# Extrinsic or Intrinsic Apoptosis by Curcumin and Light: Still a Mystery

**DOI:** 10.3390/ijms20040905

**Published:** 2019-02-19

**Authors:** Vesselina Laubach, Roland Kaufmann, August Bernd, Stefan Kippenberger, Nadja Zöller

**Affiliations:** Department of Dermatology, Venereology and Allergology, Goethe University Frankfurt, 60590 Frankfurt, Germany; v.laubach@mail.de (V.L.); kaufmann@em.uni-frankfurt.de (R.K.); august.b@web.de (A.B.); Kippenberger@em.uni-frankfurt.de (S.K.)

**Keywords:** curcumin, death receptor, apoptosis

## Abstract

Curcumin—a rhizomal phytochemical from the plant *Curcuma longa*—is well known to inhibit cell proliferation and to induce apoptosis in a broad range of cell lines. In previous studies we showed that combining low curcumin concentrations and subsequent ultraviolet A radiation (UVA) or VIS irradiation induced anti-proliferative and pro-apoptotic effects. There is still debate whether curcumin induces apoptosis via the extrinsic or the intrinsic pathway. To address this question, we investigated in three epithelial cell lines (HaCaT, A431, A549) whether the death receptors CD95, tumor necrosis factor (TNF)-receptor I and II are involved in apoptosis induced by light and curcumin. Cells were incubated with 0.25–0.5 µg/mL curcumin followed by irradiation with 1 J/cm^2^ UVA. This treatment was combined with inhibitors specific for distinct membrane-bound death receptors. After 24 h apoptosis induction was monitored by quantitative determination of cytoplasmic histone-associated-DNA-fragments. Validation of our test system showed that apoptosis induced by CH11 and TNF-α could be completely inhibited by their respective antagonists. Interestingly, apoptosis induced by curcumin/light treatment was reversed by none of the herein examined death receptor antagonists. These results indicate a mechanism of action independent from classical death receptors speaking for intrinsic activation of apoptosis. It could be speculated that a shift in cellular redox balance might prompt the pro-apoptotic processes.

## 1. Introduction

Phytochemicals have a crucial role in drug discovery and development [1,2]. Curcumin has been a part of traditional Asian medicine for thousands of years due to its extensive effects on cell physiology. It can be isolated from the rhizome of the ginger plant *Curcuma longa.* Curcumin is known for its anti-inflammatory, anti-oxidative, as well as its pro-apoptotic potential [3,4,5,6,7]. Taking into account the hardly existing toxicity of curcumin, it is predestined for the development of anti-tumorigenic therapeutic strategies. Targeting the low bioavailability of curcumin [8,9] strategies including encapsulation, inhibition of metabolic degradation and development of photodynamic therapies [6,10,11,12,13,14,15,16,17,18,19,20] have been developed. Reducing the proliferative potential of neoblastic cells as well as inducing pro-apoptotic effects is the mode of choice to target cancer cells [6,18]. These two criteria can be addressed by curcumin. Presently, there is debate whether curcumin induces apoptosis via the extrinsic or the intrinsic pathway. Treatment with high curcumin concentrations has been described to induce apoptosis depending on the cell type and tissue via the extrinsic as well as via the intrinsic pathway [21,22]. Characteristic of apoptosis induction via the extrinsic pathway is the binding of extracellular ligands to transmembrane death receptors, e.g., CD95 or tumor necrosis factor (TNF)-α receptors. Receptor clustering, binding with homologous trimeric ligands and recruitment of cytoplasmic adaptor proteins ultimately leads to auto-catalytic activation of pro-caspase-8 [23,24].

Caspase-8 thereafter cleaves and activates the effector caspases-3, -6, -7, leading to the substrate proteolysis, DNA fragmentation and cell death [24,25]. To evaluate whether curcumin in our experimental set up triggers apoptosis via the extrinsic pathway death receptor, specific antagonists were used.

## 2. Results

### 2.1. Death Receptor Specific Apoptosis Induction Was Cell Species Dependent

**Figure 1:**
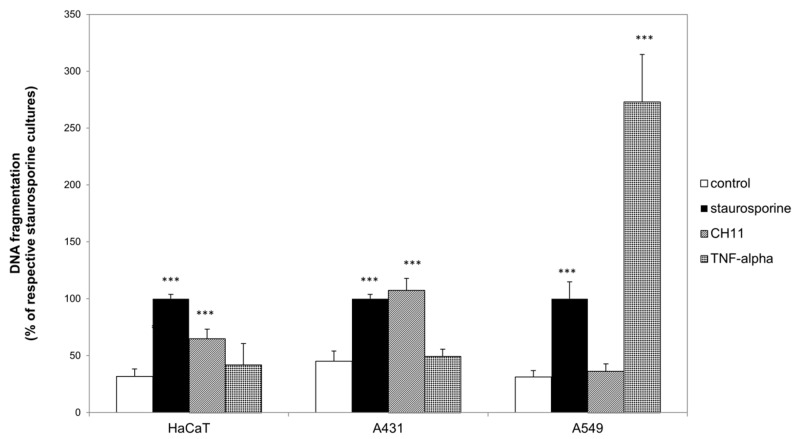
Death receptor agonist specific apoptosis induction. HaCaT, A431 and A549 cells were either left untreated (white bars) or were treated with 1 µg/mL staurosporine (black bars), with 1 µg/mL CH11 (striped bars) or 10 ng/mL tumor necrosis factor (TNF)-α (bricked bars). DNA fragmentation was evaluated after 24 h. The data displayed are representative of three experiments performed with comparable results. Average absorbance values (mean ± SD) from quintuplicate replicates per experimental condition were calculated. *** *p* ≤ 0.001 versus the respective untreated control.

**Figure 2:**
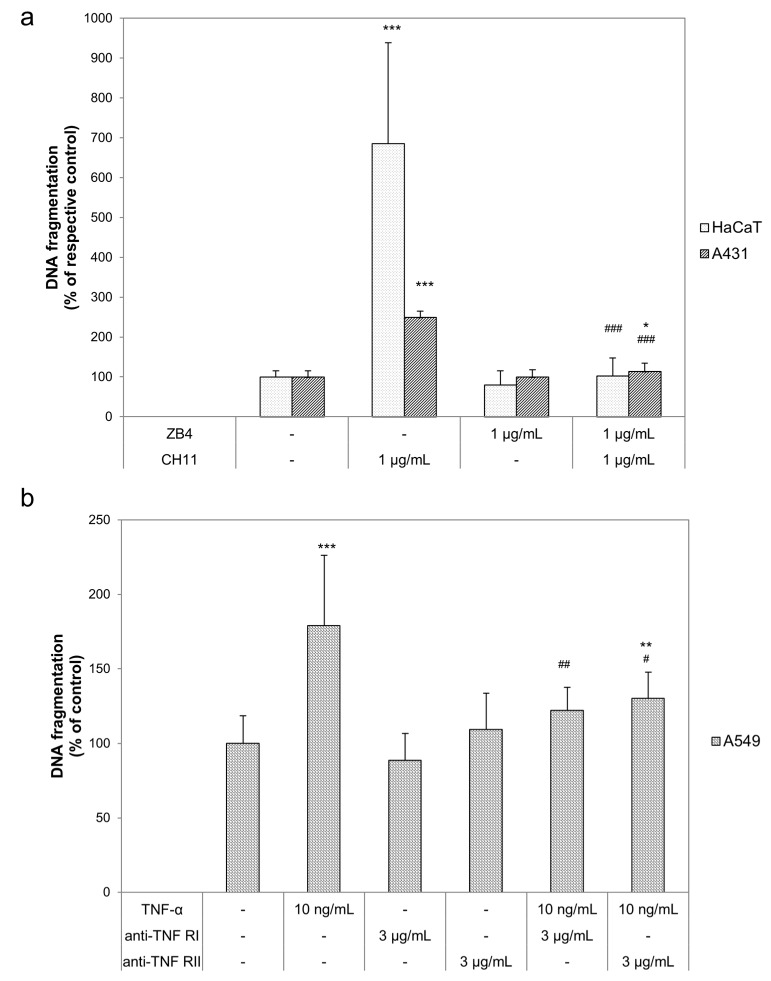
Death receptor specific antagonists reversed apoptosis induction. (**a**) HaCaT (pointed bars) and A431 (striped bars) cells were treated with 1 µg/mL CH11, 1 µg/mL ZB4 or their combination to investigate the CD95 receptor. (**b**) A549 (scaled bars) were treated with 10 ng/mL TNF-α, 3 µg/mL anti-TNF-α RI, 3 µg/mL anti-TNF-α RII or their combinations. DNA fragmentation was evaluated after 24 h. The data displayed are representative of three experiments performed with comparable results. Average absorbance values (mean ± SD) from quintuplicate replicates per experimental condition were calculated. * *p* ≤ 0.05; ** *p* ≤ 0.01; *** *p* ≤ 0.001 versus the respective untreated control and ^#^ *p* ≤ 0.05; ^##^ *p* ≤ 0.01; ^###^ *p* ≤ 0.001 versus the respective death receptor agonist.

First of all, we determined to which death receptor agonist the herein investigated epidermal cell lines are susceptible. As shown in Figure 1, DNA fragmentation was induced in all three cell lines by a positive control (1 µg/mL staurosporine; black bars) which was set to 100%. Comparison of the DNA fragmentation of the respective untreated cultures (white bars) with CH11 treated cultures (striped bars), showed significantly higher DNA fragmentation in HaCaT and A431 cells. In contrast to the observed non-inducible DNA fragmentation in A549 by CH11, TNF-α (bricked bars) induced a clear increase of DNA fragmentation in comparison to the untreated control. Neither in HaCaT nor in A431 differed DNA fragmentation of the TNF-α treated cultures from the respective untreated cultures. Therefore, we further investigated CD95 related apoptosis induction in HaCaT and A431, and TNF-α related apoptosis induction in A549. Consecutively, we tested described death receptor antagonists to compensate for the pro-apoptotic stimuli. None of the used death receptor antagonists induced DNA fragmentation (Figure 2). In combination with the respective agonists all antagonists were able to reduce the pro-apoptotic impact of the agonists. In detail ZB4 completely neutralized the pro-apoptotic influence of CH11 in HaCaT (pointed bars) and A431 (striped bars; Figure 2a). The efficiency of the two investigated TNF-α antagonists varied in A549 (scaled bars; Figure 2b). Whereas anti-TNF-α RI completely neutralized the pro-apoptotic influence of TNF-α only a decreased but still significantly higher DNA fragmentation in comparison to the untreated control was observed after treatment with TNF-α and anti-TNF-α RII.

### 2.2. Curcumin and Light Induced DNA Fragmentation Independent of the First Apoptosis Signal (FAS) Ligand and the TNF-α Receptors

**Figure 3:**
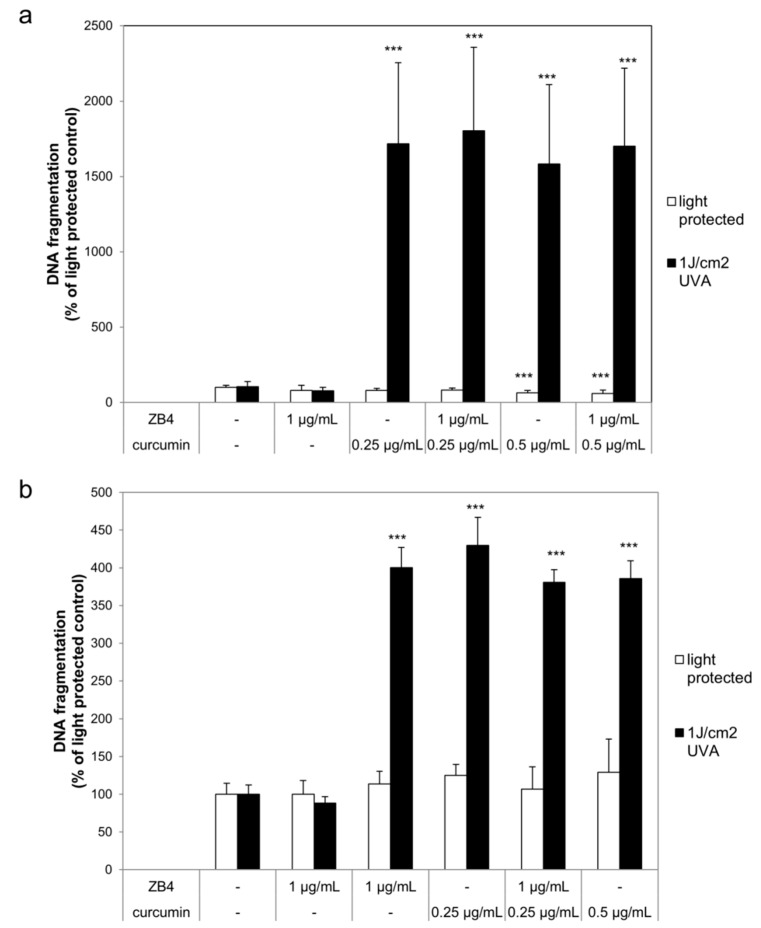
Curcumin does not induce apoptosis via CD95 HaCaT (**a**) and A431 (**b**) cells were pre-incubated with curcumin and ZB4. Thereafter the cells were irradiated with ultraviolet A radiation (UVA) followed by ZB4 exposure. DNA fragmentation was evaluated after 24 h. The applied ZB4 or curcumin concentrations had no effect on DNA fragmentation (white bars). Combining curcumin with UVA (black bars) induced significant increase of DNA fragmentation. Addition of ZB4 did not reduce the curcumin/light induced DNA fragmentation. Data displayed are representative of four experiments performed with comparable results. Average absorbance values (mean ± SD) from quadruplicate replicates per experimental condition were calculated. *** *p* ≤ 0.001 versus the respective untreated control.

**Figure 4:**
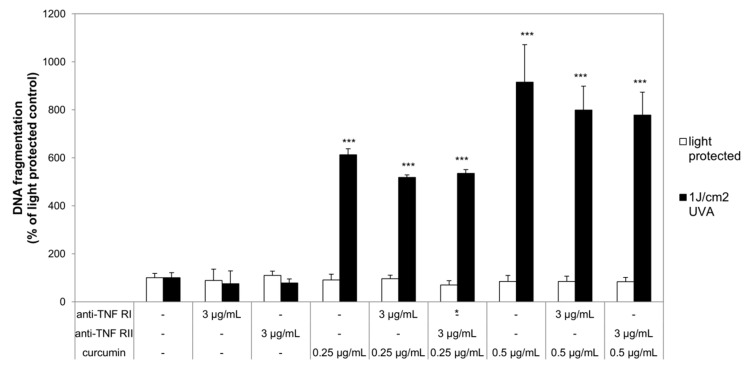
Curcumin does not induce apoptosis via TNF-α receptor I and II. A549 cells were pre-incubated with curcumin and anti-TNF-α RI or anti-TNF-α RII. Thereafter the cells were irradiated with UVA followed by anti-TNF-α RI or anti-TNF-α RII exposure. DNA fragmentation was evaluated after 24 h. The applied TNF-α receptor antagonists or curcumin concentrations had no effect on DNA fragmentation (white bars). Combining curcumin with UVA (black bars) induced a significant increase of DNA fragmentation. Neither anti-TNF-α RI nor anti-TNF-α RII reduced the curcumin/light induced DNA fragmentation. Data displayed are representative of four experiments performed with comparable results. Average absorbance values (mean ± SD) from quadruplicate replicates per experimental condition were calculated. * *p* ≤ 0.05; *** *p* ≤ 0.001 versus the respective untreated control.

After establishing the efficiency of the herein used death receptor antagonists their ability to influence DNA fragmentation in curcumin/light treated cultures was investigated. HaCaT and A431 cells were per-incubated with or without curcumin and ZB4 whereas A549 were pre-incubated with or without curcumin and anti-TNF-α RI or anti-TNF-α RII before irradiation with 1 J/cm^2^ ultraviolet A radiation (UVA). Neither curcumin nor the antagonists under light-protected conditions induced DNA fragmentation (white bars; Figure 3 and Figure 4). Likewise, the herein chosen UVA irradiation regimen did not induce significant DNA fragmentation in comparison to the light-protected controls. DNA fragmentation of cell cultures treated with curcumin and light (black bars; Figure 3 and Figure 4) was significantly increased. DNA fragmentation of HaCaT after treatment with either 0.25 µg/mL or 0.5 µg/mL curcumin and UVA was 1600% higher in comparison to the light-protected control (Figure 3a). Blocking apoptosis via the first apoptosis signal (FAS) ligand by ZB4 did not change the amount of DNA fragmentation caused by the curcumin/light treatment. Comparable results were observed in A431 cells (Figure 3b). The combined treatment of curcumin and light induced a 300% higher DNA fragmentation than observed in the light-protected cultures. As observed in HaCaT cells, addition of ZB4 to A431 cells did not influence the curcumin/light induced DNA fragmentation. In A549 cells a curcumin concentration dependent increase of DNA fragmentation after irradiation was observed (Figure 4). Cultures that had been treated with 0.25 µg/mL curcumin and light showed a 500% higher DNA fragmentation compared to the light-protected control. Increasing the curcumin concentration to 0.5 µg/mL also increased the DNA fragmentation compared to the light-protected control to 800%. Independent of the applied curcumin concentration addition of anti-TNF-α RI and anti-TNF-α, RII was not able to significantly reduce the curcumin/light induced DNA fragmentation.

### 2.3. Curcumin Increased the UVA Triggered H_2_O_2_ Generation

**Figure 5:**
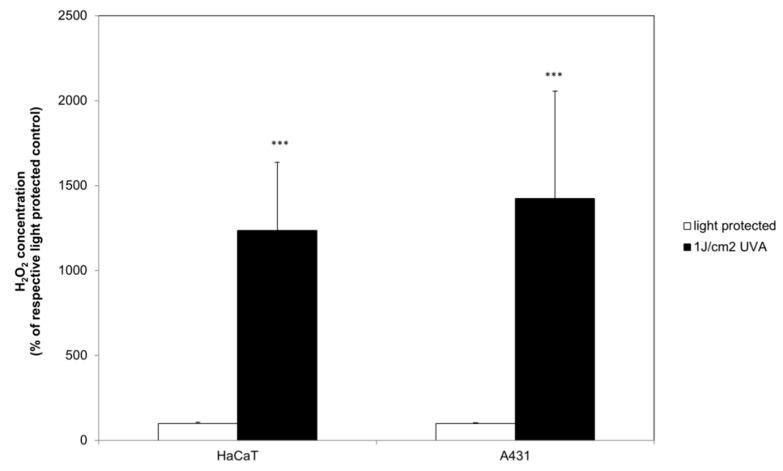
UVA induced H_2_O_2_ generation in HaCaT and A431. The data displayed are representative of four experiments performed with comparable results. Average luminescence values (mean ± SD) from triplicate replicates per experimental condition were calculated. *** *p* ≤ 0.001 versus the respective light-protected control.

**Figure 6:**
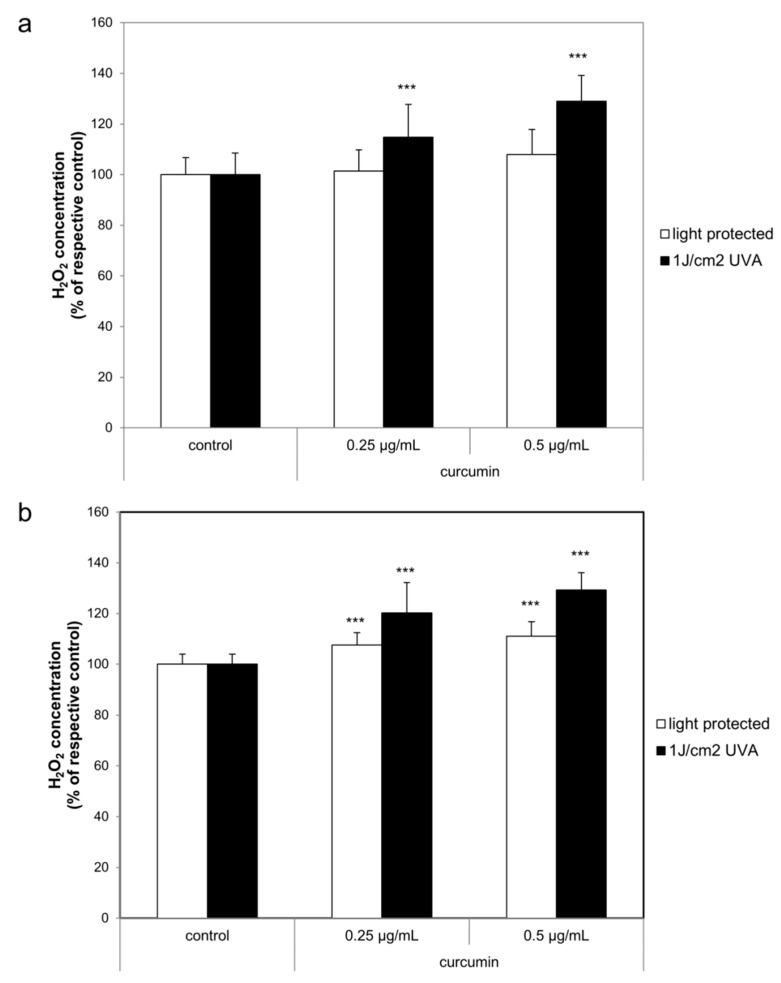
Curcumin enhanced the UVA triggered H_2_O_2_ generation in HaCaT (**a**) and A431 (**b**). The cells were pre-incubated with curcumin before irradiation with UVA. H_2_O_2_ generation was evaluated after 1 h. The data displayed are representative of four experiments performed with comparable results. Average luminescence values (mean ± SD) from triplicate replicates per experimental condition were calculated. *** *p* ≤ 0.001 versus the respective control.

After establishing that curcumin in the herein described treatment regimen does not induce apoptosis through the classical death receptors, we were interested to monitor whether the combinatorial curcumin/light treatment induces a shift of the cellular redox balance. The H_2_O_2_ concentration was measured 1 h after the treatment. Irradiation with 1 J/cm^2^ UVA induced a significant H_2_O_2_ generation increase of 1200% to 1400% in HaCaT and A431 cells (black bars, Figure 5). Under light-protected conditions (white bars) curcumin did not influence H_2_O_2_ generation in HaCaT cells (Figure 6a). Light treatment of A431 cells (Figure 6b) with curcumin induced an H_2_O_2_ increase of 7–11% in comparison to the respective controls. Comparing the UVA (black bars) induced H_2_O_2_ generation with the H_2_O_2_ generation of curcumin/light treated cultures revealed that in both cell species the H_2_O_2_ concentration was curcumin dependently increased. In HaCaT cultures treatment with 0.25 µg/mL curcumin induced a 15% higher H_2_O_2_ concentration, and treatment with 0.5 µg/mL curcumin a 29% higher H_2_O_2_ concentration than observed in the respective controls. Curcumin/light treatment of A431 cells showed a comparable influence on H_2_O_2_ generation. Treatment with 0.25 µg/mL curcumin induced a 20% increase whereas treatment with 0.5 µg/mL curcumin induced a 29% higher H_2_O_2_ concentration in comparison to the respective light-treated cultures.

## 3. Discussion

Investigation of natural compounds that have been used for centuries in traditional medicine by scientific means has increased during the last decades. Curcumin is one of those phytochemicals with an anti-cancer potential showing a lower risk of inducing adverse events [26,27] than described for other cytostatic drugs. Curcumin influences an extensive spectrum of signaling pathways involved in cancer and inflammatory diseases [28,29,30]. Establishing a photodynamic treatment combining low curcumin concentrations and radiation with either UVA or VIS [6,16,17,18] was our approach to address the low bioavailability of curcumin [9]. As it is described that curcumin interacts, e.g., with the epidermal growth factor (EGF) receptor [31,32,33], we hypothesized that curcumin also directly interacts with different death receptors facilitating apoptosis via the extrinsic pathway. There are contradictory observations concerning the mode of apoptosis induction by curcumin. On the one hand, intrinsic apoptosis induction was observed in mamma carcinoma cells as well as in HL-60 and kidney carcinoma cells [21,34,35,36,37]. On the other hand, apoptosis induction via the CD95 receptor was observed, e.g., in melanoma cells while TNF-related apoptosis-inducing ligand (TRAIL) receptor triggered apoptosis was observed in ovarial and prostate carcinoma cells [22,38,39]. In this study we analyzed whether curcumin induced apoptosis in our treatment regimen via either the CD95 or the TNF-α receptor according to Schon et al. [40]. First, we showed that susceptibility to apoptosis inductors differed in the epithelial cell lines used. In particular, HaCaT and A431 seemed to be resistant to the applied TNF-α whereas A549 did not respond to FAS ligand specific apoptosis induction. Death receptor resistance can be acquired by cells through a broad variety of modulatory mechanisms [41,42]. We were furthermore interested whether curcumin might be able to overcome such resistances. The observed curcumin/irradiation dependent induction of DNA fragmentation was taken as a positive indicator of apoptosis [4,43,44]. Hence, DNA fragmentation was monitored to observe whether the herein investigated death receptor antagonists were able to reduce or inhibit the previously described pro-apoptotic influence of the combinatory treatment with low curcumin concentrations and light irradiation. In contrast to others that observed apoptosis induction by curcumin via the FAS ligand pathway [22,45,46], no FAS ligand-related apoptosis was measured in our experimental set up. The FAS ligand specific antagonist ZB4 failed to inhibit or reduce the pro-apoptotic influence of curcumin/light treatment in either of the investigated cell lines. Furthermore, inhibition of the TNF-α receptor I and II by specific antagonists in A549 cells did not change the curcumin/light triggered apoptosis. These observations indicate that in our experimental set up, a mechanism of apoptosis induction independent from classical death receptors is very likely. A possible alternative mode of action can be related to the observations of Kim et al. [47]. They showed that curcumin-related inhibition of EGF receptor phosphorylation and subsequent inhibition of the downstream kinases lead to activation of the effector caspase-3. It seems that curcumin triggers apoptosis in a cell specific manner by different pathways. This characteristic makes curcumin potentially useful to target a broad range of different tumor cells that are sensitive to different pro-apoptotic triggers. It is known that triggering apoptosis in cells that are non-responsive to death receptor agonists or chemotherapeutics is challenging [48]. Therefore, it is of great interest to identify or develop active agents that overcome such resistances [49,50,51,52]. The observations showing that curcumin in combination with UVA-boosted H_2_O_2_ generation indicated that a shift in the cellular redox balance might elicit the observed pro-apoptotic processes as also observed by others [53,54,55,56,57,58]. Future studies need to address this issue. Moreover, utilizing more complex systems, e.g., tissue cultured skin equivalents [59,60,61] and long-term analysis are advised to further analyze the potential of curcumin to overcome chemotherapeutic resistances.

## 4. Materials and Methods

### 4.1. Cell Culture and Identification of Death Receptor Agonist Susceptibility

The spontaneous immortalized human keratinocyte cell line HaCaT [62] (kindly provided by Prof. Norbert Fusenig (German Cancer Research Institute, Heidelberg, Germany)) and the human epidermoid carcinoma cell lines A431 (ATCC^®^ CRL-1555™, American Culture Type Collection, Manassas, VA, USA) and A549 (ATCC^®^ CCL-185™, American Culture Type Collection) were cultured in Dulbecco’s Modified Eagle’s Medium (D-MEM, Gibco, Karlsruhe, Germany) with GlutaMax supplemented with 10% (*v*/*v*) fetal calf serum (FCS, PAA, Cölbe, Germany) and 1% (*v*/*v*) penicillin/streptomycin solution (Gibco) in a 7.5% CO_2_ atmosphere at 37 °C. The cells were either stimulated with 1 µg/mL staurosporine (Sigma-Aldrich, Traufkirchen, Germany) or with death receptor specific agonists: (anti-) Fas activating antibody (clone CH11, Merck Millipore, Darmstadt Germany) and TNF-α (R&D Systems, Wiesbaden-Nordenstadt, Germany). These death receptor agonists were combined with their respective antagonists: anti-Fas neutralizing antibody (clone ZB4, Merck Millipore) and anti-TNFα RI and anti-TNFα RII (both R&D Systems).

### 4.2. Irradiation Regimen

Curcumin (Sigma-Aldrich) was dissolved and applied as previously described [16,17]. Briefly, cells were incubated for 1 h in a medium containing 0.25–0.5 µg/mL curcumin, with or without the above mentioned death receptor antagonists. After replacement of the culture medium with PBS^++^ (Gibco) the cells were either kept light, and/or were irradiated with 1 J/cm^2^ ultraviolet A (UVA, Waldmann, Villingen-Schwenningen, Germany). After irradiation PBS^++^ was replaced with culture medium with and without the above mentioned death receptor antagonists.

### 4.3. DNA Fragmentation

DNA fragmentation, the chosen apoptosis indicator, was quantified after 24 h. The adherent cells were lysed and DNA fragmentation was analyzed with the Cell Death Detection (CDD; Roche, Mannheim, Germany) enzyme-linked immunosorbent assay (ELISA), as described [16].

### 4.4. Monitoring of the Cellular Redox Balence

The generation of H_2_O_2_—monitored with the ROS-Glo™-H_2_O_2_ Assay (Promega, Mannheim, Germany) was chosen as the indicator for oxidative stress in relation to the herein described treatment regimen. Briefly, cells were pre-incubated for 1 h with PBS^++^ containing 0.25–0.5 µg/mL curcumin and were subsequently irradiated with 1 J/cm^2^ UVA. After 1 h the assay was conducted as recommended by the manufacturer. The luminescence was recorded using a microplate luminometer (CentroPro LB962, Berthold Technologies, Bad Wildbach, Germany).

### 4.5. Presentation of Data and Statistical Analysis

All data are presented as mean values ± standard deviation. Statistical significance of the data was evaluated by the Wilcoxon-Mann-Whitney *U*-test (BiAS, version 11.06, epsilon-Verlag, Frankfurt, Germany). Each set of data was related to the referring untreated control (*) or the respective agonist (#). Differences were considered significant at *^,#^ *p* ≤ 0.05; **^,##^ *p* ≤ 0.01; ***^,###^ *p* ≤ 0.001.

## Abbreviations

TNF-α	tumor necrosis factor α
UVA	ultraviolet A
EGF	endothelial growth factor
